# Project FRONTIER: a Cohort to Study Aging in Rural West Texas

**DOI:** 10.1002/alz.095032

**Published:** 2025-01-09

**Authors:** Boris Decourt, Jonathan Singer, Veronica Molinar‐Lopez, XiangLing Yin, Volker Neugebauer

**Affiliations:** ^1^ Texas Tech University Health Sciences Center, Lubbock, TX USA; ^2^ Texas Tech University, Lubbock, TX USA

## Abstract

**Background:**

In the past decades, the NIH has identified a large knowledge gap in the study of health and disease in diverse and underserved inhabitants. The most recent estimates (2020) indicate that rural dwellers represent 15‐20% of the total US population, but these individuals experience worse health status than urban counterparts. A major challenge to the study of health disparities in rural areas is the dearth of structured rural cohorts that can be used for scientific research purposes.

**Method:**

Project FRONTIER (Facing Rural Obstacles to Healthcare Now Through Intervention, Education & Research) was initiated in 2006 as an epidemiological study to explore the natural course of chronic disease development and its impact on longitudinal cognitive, physical, social and interpersonal functioning in a multi‐ethnic adult sample from rural communities of West Texas.

**Result:**

Currently, project FRONTIER is collecting data from Cochran, Bailey, Parmer, Hockley and Lubbock counties, with expansion to additional counties soon [update: Lamesa/Dawson county]. In recent years, the cohort has been utilized more for cognitive and Alzheimer’s disease research than other conditions, though we collected limited data and biospecimens. Thus, there is a huge opportunity to better characterize this cohort at the genetic and metabolic levels to gain large amounts of information on the aging processes in rural dwellers. The demographics of this cohort are: 1374 unique individuals, 66% female, 56% Hispanics, mean age of 58.9±12.2 years, 21% with a high school degree, and 23% with some college education. Although challenging, we are attempting longitudinal collections on this cohort. However, as our group is recruiting more experts on rural cohort research, we are currently implementing more rigorous protocols to collect accurate data and high quality biospecimens more frequently from this cohort.

**Conclusion:**

Our Project FRONTIER cohort provides very informative data on dementia risk factors in rural dwellers. As we make progress, we intend to share these materials with the scientific community for the deep genotyping and phenotyping of our study participants.

